# Occurrence of autochthonous neurocysticercosis in Germany: a case report

**DOI:** 10.1186/s12879-026-12859-w

**Published:** 2026-02-11

**Authors:** Franziska Lordick, Henning Trawinski, Christoph Lübbert

**Affiliations:** 1 Division of Infectious Diseases and Tropical Medicine, Department of Medicine I, Leipzig University Medical Center, Leipzig, Germany; 2 Interdisciplinary Center for Infectious Diseases (ZINF), Leipzig University Medical Center, Leipzig, Germany; 3 Department of Infectious Diseases and Tropical Medicine, Hospital St. Georg, Leipzig, Germany

**Keywords:** Neurocysticercosis, *Taenia solium*, Pork tapeworm, Autochthonous, Local transmission, Europe, Case report

## Abstract

**Background:**

Neurocysticercosis [NCC] represents a severe manifestation of parasitic infection of the central nervous system [CNS] by larvae of *Taenia (T.) solium*, the pork tapeworm. Although it is one of the main causes of acquired epilepsy worldwide, endemic transmission in Europe has been largely eliminated, with only isolated autochthonous cases occurring.

**Case presentation:**

A 43-year-old woman from a rural region in Saxony-Anhalt, Eastern Germany, with no history of travel abroad or contact with livestock or known tapeworm carriers presented with a first-time generalized epileptic seizure. Cranial magnetic resonance imaging [cMRI] showed multiple ring-enhancing lesions with surrounding edema and calcifications. Histopathological examination of a brain biopsy raised suspicion of a parasitic infection based on granulomatous inflammation with eosinophilic infiltrates, giant cells, and amorphous material. Subsequent molecular analysis using real-time polymerase chain reaction [PCR] detected *T. solium* DNA. Neoplasms and alternative (granulomatous) diseases were excluded. Serological testing and stool microscopy for *T. solium* eggs were negative in both the patient and her immediate family members. The definitive diagnosis of NCC was established. Antiparasitic treatment with albendazole and praziquantel in combination with corticosteroids was initiated and anticonvulsive therapy was continued. The patient remained seizure-free during follow-up, and repeated cMRI showed significant regression of the cerebral lesions.

**Conclusions:**

This case demonstrates a rare case of highly suggestive autochthonous NCC in Western Europe and highlights the diagnostic challenges in non-endemic regions, as well as the importance of considering NCC in the differential diagnosis of patients presenting with typical symptoms such as seizures and NCC-compatible findings in neuroimaging, even if the medical history does not suggest transmission of *T. solium*. Strengthening awareness among physicians and improving diagnostic tools for direct pathogen detection are crucial to enable timely diagnosis even in cases of negative serology.

**Clinical trial number:**

Not applicable.

**Supplementary Information:**

The online version contains supplementary material available at 10.1186/s12879-026-12859-w.

## Background

The zoonotic cestode *Taenia (T.) solium* has a life cycle involving humans, pigs and environmental contamination with eggs. In humans, infection can cause intestinal taeniasis and cysticercosis. The latter refers to a systemic infection with involvement of various organs, most notably the central nervous system [CNS], known as neurocysticercosis [NCC]. Extraneural manifestations, such as ocular, muscular, or cardiac cysticercosis may also occur [1].

**Fig. 1:**
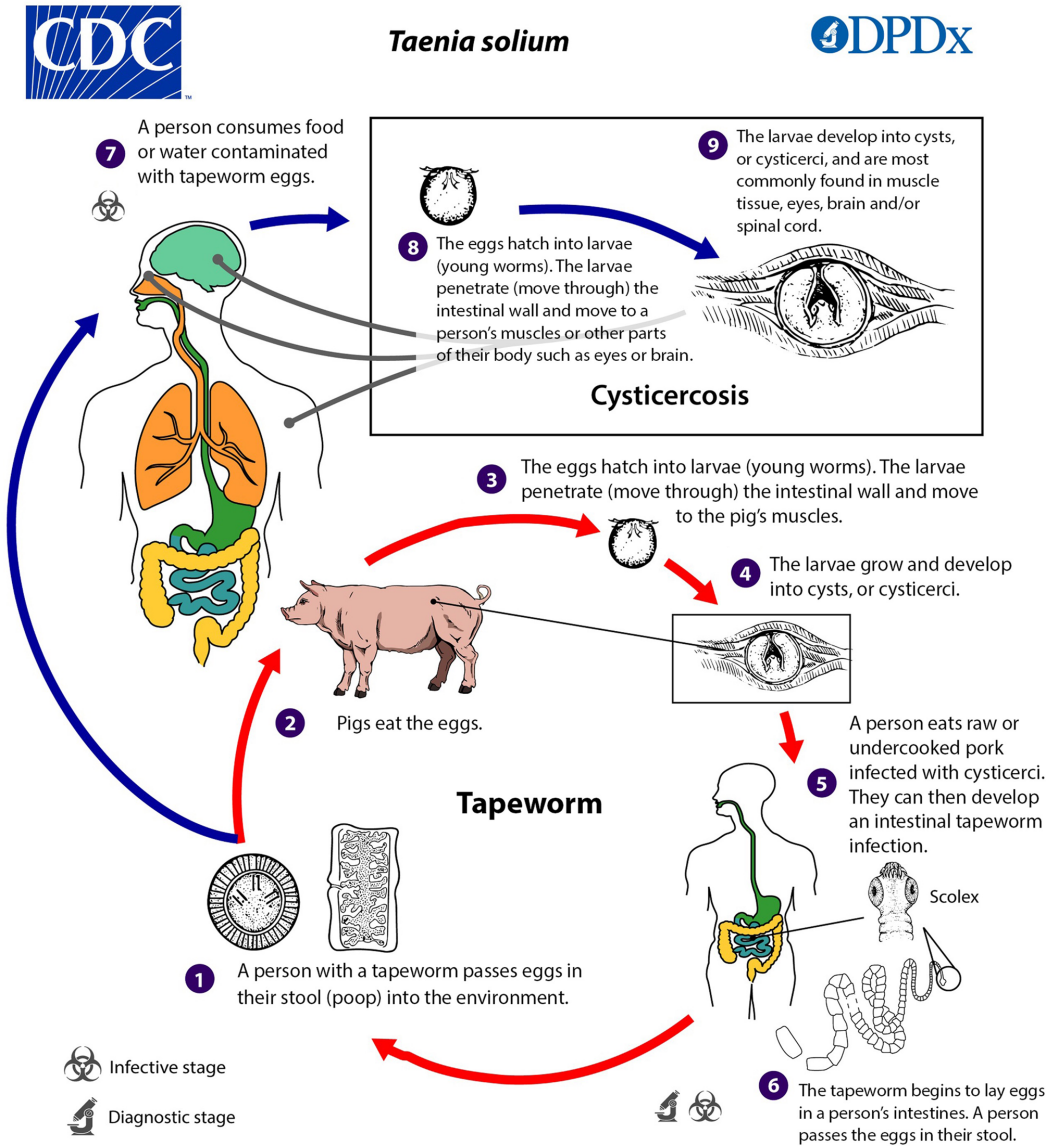
Illustration depicting the life cycle of *T. solium*. CDC Division of Parasitic Diseases and Malaria (DPDx), Centers for Disease Control and Prevention, Public Domain [25]

Taeniasis is acquired through consumption of undercooked pork meat containing cysticerci, the larval stage of *T. solium*. Within the human intestine, cysticerci mature into adult tapeworms. This condition is usually asymptomatic or associated with mild gastrointestinal symptoms. Adult tapeworms produce eggs which are excreted in human feces, resulting in environmental contamination. Humans acquire cysticercosis through fecal-oral ingestion of embryonated *T. solium* eggs containing oncospheres via contaminated food or water, poor hand hygiene, or autoinfection in tapeworm carriers. After intestinal penetration, hematogenous dissemination of oncospheres can result in cyst development within the CNS, particularly in the brain parenchyma, subarachnoidal space, ventricles, and the spinal cord, causing NCC [2] (Fig. 1).

Clinical manifestations vary depending on cyst location, size, and developmental stage. Most commonly, patients present with epileptic seizures, headache, focal neurological deficits, or hydrocephalus. However, a latency period of months to decades between infection and the onset of symptoms is typical, and some individuals remain asymptomatic [1].

The geographical distribution of NCC is concentrated in areas with poor hygiene, inadequate sanitation, and pig husbandry practices. NCC accounts for nearly one-third of epilepsy cases in highly endemic tropical and subtropical areas, particularly in Latin America and sub-Saharan Africa [3, 4]. Prior to the implementation of control measures in the 1990s, endemic transmission also occurred in Southern and Eastern Europe, most notably in Portugal and Spain [5]. Due to the lack of specific surveillance systems, the current extent of NCC occurrence in Europe is not easily assessable. Most cases are related to travel and migration, but sporadic autochthonous transmission still occurs. Between 2000 and 2019, fewer than 30 autochthonous cases of NCC were reported in European Union member states and associated countries, with more than half of these cases originating from Eastern Europe, such as Romania and Serbia [6, 7].

The diagnosis of NCC is based on histological demonstration of the parasite or characteristic neuroimaging findings, in combination with typical clinical manifestations and epidemiological or exposure evidence, while serological tests (enzyme-linked immunoelectrotransfer blot [EITB], enzyme-linked immunoassay [ELISA]) provide supportive information. It is essential to rule out other causes of cystic lesions (e.g. neoplasm, tuberculoma), so histopathological examination of biopsy samples may be imperative [8, 9].

Antiparasitic treatment options include a one- or two-week course of albendazole, combined with praziquantel in more severe cases, and administered in combination with corticosteroids to prevent worsening of the inflammation. For seizure control, antiepileptic drugs should be complemented [8].

## Case presentation

In July 2024, a 43-year-old female patient from a rural area in Saxony-Anhalt, Eastern Germany, presented to a regional hospital after suffering a first-time unprovoked generalized epileptic seizure with loss of consciousness. She reported no previous focal neurological deficits or headaches. The family members who shared the same household were asymptomatic. As a saleswoman at a highway service station, she had frequent contact with other people on a daily basis. No stays outside of Germany were reported in the past. Contact or close coexistence with farm animals was denied.

**Fig. 2:**
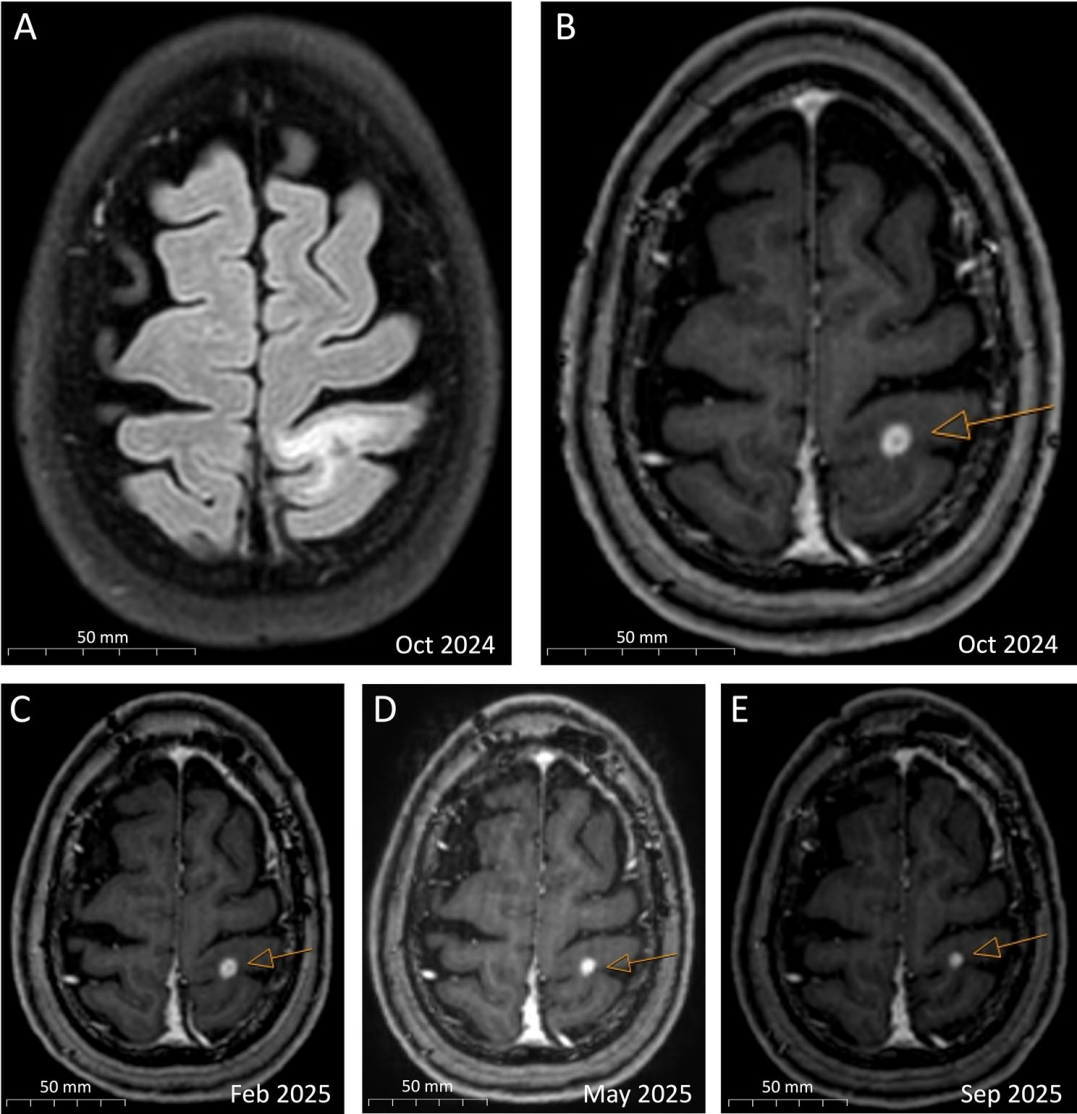
cMRI scans showing a ring-enhancing lesion (orange arrows) with surrounding edema in the left postcentral gyrus, decreasing in size from 8 mm to 4 mm within a 12-month time span. **A**: cMRI scan, axial plane, FLAIR. **B–E**: cMRI scans, T1 weighted, axial plane, contrast enhanced

**Fig. 3:**
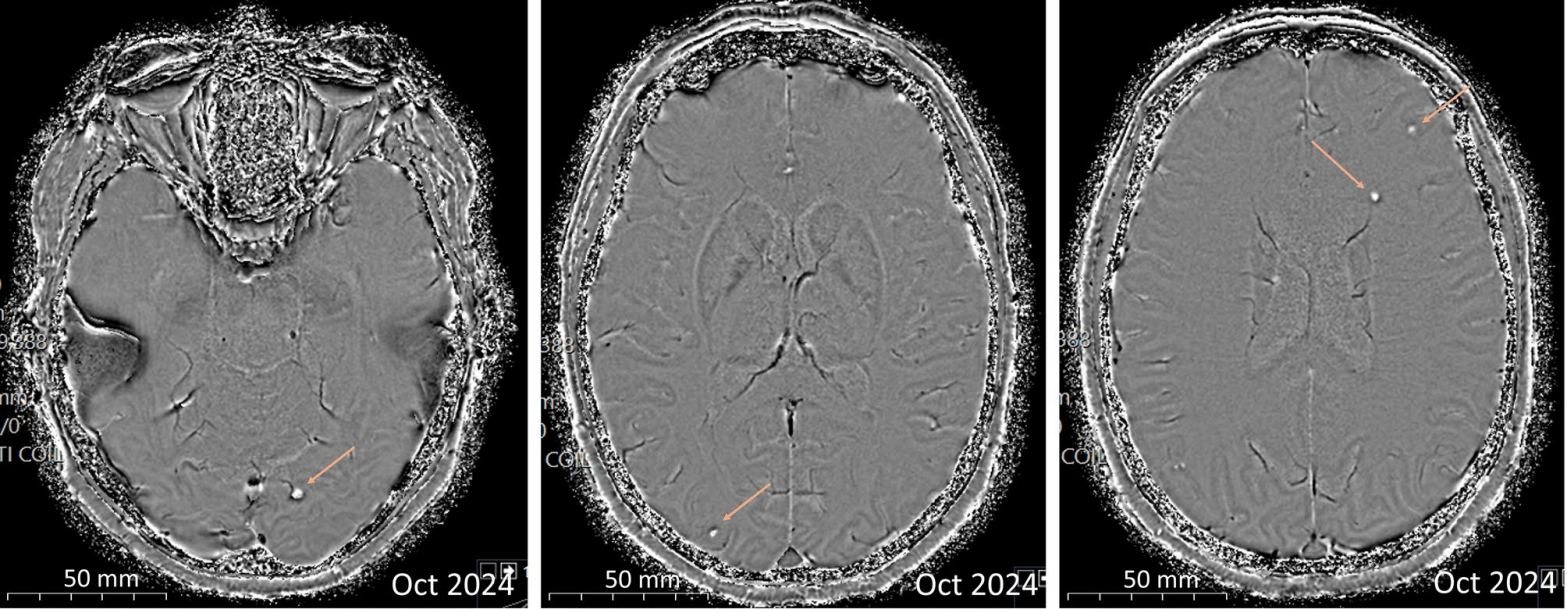
cMRI scans showing parenchymal calcifications (orange arrows), axial plain, SWI

Laboratory findings in blood and cerebrospinal fluid [CSF] were unremarkable except for slightly elevated C-reactive protein [CRP] serum levels (21.3 mg/L), as summarized in Additional file 1: Table S1. No abnormalities were found in the differential blood count, including normal eosinophil levels (0.3/nL). Cranial MRI [cMRI] was initially misinterpreted as cerebral metastases, showing a bihemispheric pattern of eight small (3–8 mm), ring-enhancing lesions with surrounding white matter edema without mass effect (Fig. 2A and B). Multiple small intracerebral calcifications were also observed (Fig. 3). A comprehensive evaluation, including an FDG-PET/CT scan, revealed no evidence of a primary tumor or metastases. HIV infection was ruled out in view of potential differential diagnoses such as cerebral toxoplasmosis. Serological testing for toxoplasmosis and syphilis were negative, and CSF analysis for *Toxoplasma* DNA and cryptococcal antigens was unremarkable.

**Fig. 4:**
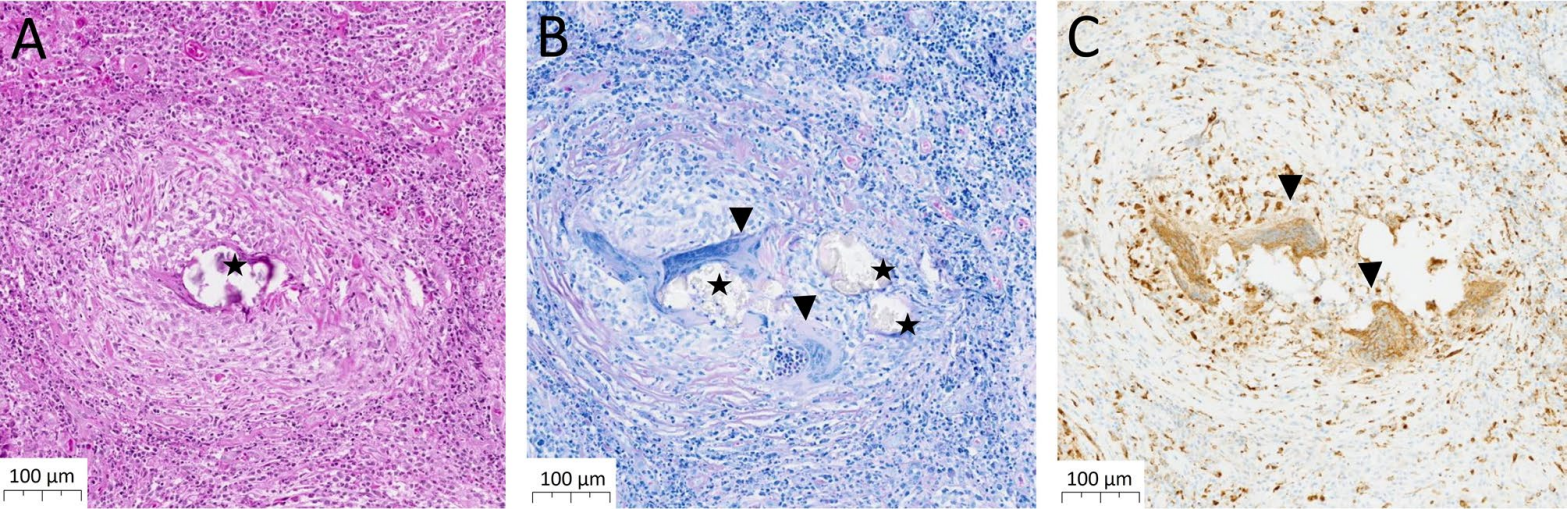
Representative images of the brain lesion showing a granulomatous inflammatory reaction. **A**: Hematoxylin and eosin staining reveals a granulomatous inflammatory reaction with presumed parasitic remnants in the center (star). **B**: Giemsa staining highlights the parasitic larvae (star) and the surrounding giant cell reaction (triangle). **C**: Immunohistochemical CD68 staining marks multinucleated giant cells (triangle) and the associated macrophages within the granuloma. Scale bar: 100 μm

Histopathological examination of a brain biopsy taken from the left frontal region revealed no evidence of malignancy, but showed a granulomatous inflammation with eosinophilic infiltrates, foreign-body giant cells, and amorphous material suggestive of parasitic remnants (Fig. 4). No fungal or other pathogenic elements were identified. Sarcoidosis and tuberculosis were largely excluded based on granuloma morphology and negative Ziehl-Neelsen staining, with simultaneous negative mycobacterial culture and PCR from CSF.

Given the suspicion of a parasitic infection, molecular testing was requested from the German National Reference Center for Tropical Pathogens (Bernhard Nocht Institute, Hamburg, Germany). Although no intact parasitic structures were identified, *T. solium* DNA (cycle threshold [C_t_] value 39.03) was detected in formalin-fixed tissue sections using pTsol9-specific DNA-based PCR. *Echinococcus* and nematode DNA could not be detected. The EITB assay performed simultaneously using lentil lectin-purified glycoprotein extracts to detect specific antibodies for *T. solium* antigens and serological follow-up tests remained negative. Neither antigen-detection assays nor serology or PCR of CSF were performed. Microscopic examination of the patient’s stool revealed no evidence of helminth eggs. Further diagnostic evaluation ruled out eye involvement by means of fundoscopy. Examination of family members for tapeworm carriage, including microscopic stool examinations and *T. solium* serology, was unremarkable.

Based on the internationally recognized diagnostic criteria proposed by Del Brutto et al., this case fulfills two major neuroimaging criteria (enhancing lesions; typical parenchymal brain calcifications) along with seizures as a clinical criterion, allowing a definitive diagnosis of NCC. The diagnosis is further supported by histopathological and molecular pathological findings, as well as a thorough exclusion of alternative differential diagnoses [9].

In accordance with current guidelines, weight-adjusted anthelminthic treatment with albendazole (15 mg/kg/day in three doses) and praziquantel (50 mg/kg/day in three doses) was initiated on an inpatient basis and continued for two weeks, alongside concomitant anti-inflammatory therapy with prednisolone (1 mg/kg/day) which was gradually tapered. The already established anticonvulsive therapy with levetiracetam was maintained [8].

During regular clinical follow-up examinations, the patient remained asymptomatic and had no further epileptic seizures. An initially abnormal EEG which indicated regional cerebral dysfunction in the right parietal area normalized over time. Follow-up cMRI showed a general reduction in cerebral lesions (Fig. 2B to E), with complete regression observed in only one lesion located in the left frontal gyrus.

### Discussion and conclusion

We presented one of the very rare highly suggestive autochthonous NCC cases in Europe, involving a woman from a rural area in Eastern Germany with a typical clinical presentation and no history of travel to endemic areas.

Since 2000, only six cases of *T. solium* NCC have been published in Germany [10]. Among these, only two patients had no known exposure to endemic settings: a 69-year-old male who spent his childhood on a pig farm and suffered his first epileptic seizure in 2009, and, five years later, a 75-year-old female with stroke-like symptoms [11, 12]. Although autochthonous transmission in these cases is conceivable, infection acquired decades earlier, when *T. solium* was still largely endemic in Europe, cannot be ruled out. Similarly, the absence of travel in our patient makes autochthonous acquisition highly suggestive. However, the long latency period of NCC also opens up the possibility that our patient may have been infected during childhood in a region of Germany where the disease was endemic at that time.

It should also be noted that cases of NCC due to other *Taenia* species, which are endemic in temperate regions, have rarely been reported in Europe. For instance, infection by the canid tapeworm *T. crassiceps* in two patients from Southern Germany showed cerebral involvement that was clinically and radiologically indistinguishable from NCC caused by *T. solium* [13, 14]. This highlights the importance of molecular pathogen identification, particularly with regard to epidemiological context [15].

Our case emphasizes the diagnostic challenges of NCC in non-endemic regions. Transmission by often asymptomatic carriers of *T. solium* tapeworms, the typically prolonged onset of symptoms, and the absence of pathognomonic clinical features increase the risk of diagnostic delay and misdiagnosis. Laboratory findings are generally nonspecific and depend on the stage of the disease and cyst location. As shown in our case, eosinophil counts are not a reliable indicator of NCC [1, 16].

Neuroimaging findings in non-endemic regions often raise suspicion of malignancy, given its much higher prevalence compared to parasitic infections. NCC can present as single or multiple enhancing or ring-enhancing nodules, or mass-like lesions with surrounding edema, closely mimicking brain metastases, gliomas, or lymphomas. The absence of uncommon pathognomonic findings, such as a visible scolex, further complicates differentiation [1, 9]. MRI remains the imaging modality of choice for the assessment of parenchymal viable disease, parenchymal granulomas, and extraparenchymal NCC, whereas computed tomography is particularly valuable for the identification of calcified lesions or when MRI is unavailable or contraindicated [8].

Serological testing is a valuable supportive diagnostic tool. While antibody-based assays, such as EITB, show high sensitivity in patients with multiple viable cysts, antibody production may not be detectable in cases with single or calcified lesions or intraparenchymal location, as observed in our patient. Approximately 30% to 50% of patients with NCC remain seronegative. Moreover, EITB cannot reliably distinguish active from past infection due to antibody persistence [1, 8, 9]. Antigen-detection assays, although less widely available, address this limitation by indicating the presence of viable cysticerci and are particularly useful for guiding treatment decisions [9, 17]. Broader access to validated antigen-based or point-of-care assays may improve diagnostic accuracy, prevent unnecessary antiparasitic treatment and strengthen clinical surveillance.

PCR on biopsy specimens is highly sensitive and specific, providing a reliable confirmatory tool in tissue samples, although it is rarely required when histology is available. In our case, *T. solium* DNA was detected in the formalin-fixed biopsy specimen, despite the absence of intact parasitic structures. The relatively high C_t_ value is likely due to the damaging effect of formalin on DNA, which might significantly reduce PCR amplification efficiency [18]. Additionally, parasite load in necrotic or degenerating lesions is usually low, which cannot be excluded in this case [1]. In comparison, sensitivity of PCR in CSF is lower and depends on cyst location, with the highest performance observed in extraparenchymal NCC [19]. A study from Ecuador showed that serological findings and PCR should be used complementarily; Romo et al. found the following assay sensitivities: HP10 Ag in serum (41.7%, 95% confidence interval [CI] 22.1–63.4), HP10 Ag in CSF (87.5%, 95% CI 67.6–97.3), and PCR in CSF (79.2%, 95% CI 57.9–92.9) [20].

Therapeutic management must be individualized based on clinical and neuroimaging findings. Antiparasitic therapy should be initiated only under corticosteroid coverage to mitigate inflammatory complications. It is not indicated in the absence of viable parasites, such as in calcified lesions. In our patient, neuroimaging demonstrated a mixed pattern with degenerating lesions alongside more than two viable cysts. In accordance with current guidelines, dual antiparasitic therapy was initiated [8, 16].

The involvement of various specialties, including infectious diseases, radiology, neurology, and ophthalmology, underscores the need for multidisciplinary expertise in managing NCC [5]. Targeted education and training for healthcare professionals is crucial, particularly in non-endemic regions [6]. The diagnostic criteria proposed by Del Brutto et al. aim to standardize the diagnostic approach: a definitive diagnosis of NCC is based either on an absolute criterion (histological parasite identification; subretinal cysticercus; scolex on neuroimaging) or on the accumulation of robust indirect evidence, including neuroimaging. Complementary investigations are primarily intended to exclude alternative pathologies when imaging findings are inconclusive. Prior to invasive diagnostic procedures such as surgical biopsy, thorough history-taking, serological testing, and assessment of close contacts for tapeworm carriage are recommended. Systematic screening of the patient’s close environment remains an important public health measure to identify potential sources and prevent further transmission [8, 9].

The occurrence of autochthonous NCC in industrialized countries raises questions regarding potential routes of transmission. Outbreaks in non-endemic communities, such as the spread among orthodox Jews in New York City or, more recently, among schoolchildren in Belgium [21, 22], are most plausibly explained by fecal-oral transmission from asymptomatic tapeworm carriers who acquired the infection in endemic regions and subsequently introduced it into non-endemic settings [23]. Other transmission routes include the consumption of insufficiently washed fruit and vegetables potentially contaminated with *T. solium* eggs, particularly when fertilized with human feces [1, 8]. While rural living and exposure to contaminated environments are recognized risk factors in endemic areas, conclusions regarding local transmission in Germany should be interpreted cautiously, as this represents an isolated case [24]. However, the growing trend toward organic pork production in Europe through free-range pig farming and home slaughtering without adequate veterinary supervision, poses a potential challenge to the control of cysticercosis [5]. In our patient, transmission may have occurred during occupational activities at the highway service station, e.g. through frequent contact with long-distance travelers from potentially endemic regions. However, this remains a speculative hypothesis without supporting epidemiological data. Future systematic studies could help to assess whether occupational contact with travelers or migrants in non-endemic settings is associated with an increased risk of acquiring rare imported infectious diseases such as NCC.

In conclusion, our case emphasizes the importance of considering NCC in patients presenting with neurological symptoms and compatible neuroimaging findings, even without exposure to endemic areas. Increasing physician awareness, expanding access to reliable diagnostic tools, and strengthening clinical surveillance are crucial to ensure timely diagnosis, effective treatment, and prevention of potential local transmission.

## Supplementary Information

Below is the link to the electronic supplementary material.

Supplementary Material 1

Supplementary Material 2

## Data Availability

The datasets generated and analyzed during the current study are not publicly available as they contain sensitive personal data, but are available from the corresponding author on reasonable request.

## Abbreviations

cMRI: Cranial magnetic resonance imaging
CNS: Central nervous system
CRP: C-reactive protein
CSF: Cerebrospinal fluid
C_t_: Cycle threshold
DNA: Deoxyribonucleic acid
EEG: Electroencephalography
EITB: Enzyme-linked immunoelectrotransfer blot
ELISA: Enzyme-linked immunoassay
FDG-PET/CT: Fluorodeoxyglucose positron emission tomography/computed tomography
FLAIR: Fluid attenuated inversion recovery
NCC: Neurocysticercosis
PCR: Polymerase chain reaction
SWI: Susceptibility-weighted images
