# Supplementary material for: Occurrence of autochthonous neurocysticercosis in Germany: a case report
Source: BMC Infect Dis. 2026 Feb 11;26:571. doi: 10.1186/s12879-026-12859-w (PMC12997914; doi:10.1186/s12879-026-12859-w)
Supplement: Supplementary file 2 — Supplementary Material 2 [file 12879_2026_12859_MOESM2_ESM.pdf]

**Additional file 1: Table S1.** Laboratory findings from 07.07.2024.

| Test | Value | Unit | Reference range |
|---|---|---|---|
| **Hematology** | | | |
| Hemoglobin (Hb) | 8.2 | mmol/l | 6.7 - 9.7 |
| Hematokrit (Hct) | 0.4 | l/l | 0.35 - 0.4 |
| Red blood cell count (RBC) | 4.0 | $\times 10^{12}$/l | 4.1 - 5.1 |
| White blood cell count (WBC) | 11.2 | $\times 10^{9}$/l | 4.4 - 11.3 |
| Platelets | 274 | $\times 10^{9}$/l | 150 - 500 |
| **Differential blood count** | | | |
| Neutrophils | 69.8 | % | 37.0 - 80.0 |
| Lymphocytes | 21.9 | % | 15.0 - 50.0 |
| Monocytes | 7.1 | % | 0.0 - 10.0 |
| Eosinophils | 1.0 | % | 0.0 - 7.0 |
| Basophils | 0.2 | % | 0.0 - 2.5 |
| **Coagulation** | | | |
| Prothrombin time (PT) | 117 | % | 70 - 140 |
| International Normalized Ratio (INR) | 0,90 | | 0.90 - 1.4 |
| Activated Partial Thromboplastin Time (aPTT) | 24 | s | < 40 |
| **Biochemistry** | | | |
| Sodium ($Na^+$) | 138 | mmol/l | 136 - 145 |
| Potassium ($K^+$) | 3.9 | mmol/l | 3.5 - 5.1 |
| Calcium total ($Ca^{2+}$) | 2.28 | mmol/l | 2.1 - 2.55 |
| Creatinine | 72 | µmol/l | 50 - 98 |
| Urea | 3.2 | mmol/l | 2.5 - 6.7 |
| Glomerular Filtration Rate (GFR) | 81.3 | mL/min/1.73 $m^2$ | 50 - 102 |
| C-Reactive Protein (CRP) | 21.3 | mg/l | < 5 |
| Lactate Dehydrogenase (LDH) | 3.28 | µkat/l | 2.08 - 3.6 |
| Glucose | 6.4 | mmol/l | 3.9 - 5.8 |
| Aspartate Aminotransferase (AST) | 0.31 | µkat/l | 0 - 0.52 |
| Alanine Aminotransferase (ALT) | 0.23 | µkat/l | 0 - 0.57 |
| Gamma-GT | 0.50 | µkat/l | 0 - 0.67 |
| **Cerebrospinal fluid** | | | |
| White blood cells (WBC) | 1.38 | /µl | 0 - 3 |
| Glucose | 3.30 | mmol/l | 2.2 - 3.8 |
| Lactate | 1.89 | mmol/l | 1.1 - 2.4 |
| Total protein | 402 | mg/l | 150 - 400 |
